# Postoperative recurrence of clinical early-stage non-small cell lung cancers: a comparison between solid and subsolid nodules

**DOI:** 10.1186/s40644-019-0219-3

**Published:** 2019-06-07

**Authors:** Shingo Iwano, Hiroyasu Umakoshi, Shinichiro Kamiya, Kohei Yokoi, Koji Kawaguchi, Takayuki Fukui, Shinji Naganawa

**Affiliations:** 1 Department of Radiology, Nagoya University Graduate School of Medicine, 65 Tsurumai-cho, Showa-ku, Nagoya, 466-8550 Japan; 2 Department of Thoracic Surgery, Nagoya University Graduate School of Medicine, Nagoya, Japan; 3 Department of Radiology, Toyohashi Municipal Hospital, Toyohashi, Japan

**Keywords:** Non-small cell lung cancer, Computed tomography, Prognostic factors, TNM classification, Solid size

## Abstract

**Background:**

For subsolid non-small cell lung cancers (NSCLCs), solid size (SS), which is the maximal diameter of the solid component, correlates more accurately with tumor prognosis than the total size, which is the maximal diameter of the entire tumor, including ground-glass opacity. We reviewed the propriety of the TNM staging based on the SS for early-stage NSCLCs.

**Methods:**

We retrospectively reviewed the preoperative radiological reports, clinical records, and pathological reports of NSCLC cases in our hospital between 2010 and 2013, and clinical stage (c-Stage) 0 and I tumors were selected. Disease-free survival (DFS), based on survival analysis, was used to assess the tumor characteristics that predicted the prognosis.

**Results:**

A total of 247 NSCLC diagnoses in 231 patients (88 women and 143 men; age, 67 ± 7 years) were included in our cohort. They were classified into solid (*n* = 131) and subsolid (*n* = 116) nodules. The DFS curves indicated that prognosis was significantly worse in the following order: c-Stage 0, c-Stage IA, and c-Stage IB tumors (*p* = 0.016). Patients with solid nodules showed a significantly worse prognosis than patients with subsolid nodules (*p* < 0.001). A multivariate Cox proportional hazards model showed that the significant predictive factors for DFS were c-Stage (hazard ratio, 1.600; *p* = 0.020) and solid nodules (hazard ratio, 3.077; *p* = 0.031).

**Conclusions:**

For early-stage NSCLCs, the c-Stage based on the SS in subsolid nodules was useful for predicting postoperative DFS. In addition, whether nodules were solid or subsolid was another independent prognostic factor.

## Background

It is well known that the prognosis of non-small cell lung cancer (NSCLC) with masses equal to or less than 3 cm in diameter is favorable [1, 2]. Therefore, localized tumors that meet this criteria on computed tomography (CT) images without nodes or distant metastases are considered early-stage cancers (clinical T1aN0M0; stage IA), according to the tumor–node–metastasis (TNM) classification of the Union for International Cancer Control (UICC) [3].

NSCLCs are classified clinically into solid nodules, including only solid components, and subsolid nodules, including ground-glass opacity (GGO) components, based on thin-section CT findings. Some previous studies have focused on the solid component that reflects the intra-tumoral collapse of the airspace or fibrosis within the subsolid nodules and have demonstrated that the solid size (SS), which is the maximal diameter of the solid component, correlates with tumor invasiveness and patient prognosis [4–7]. Therefore, SS measurements have been adopted as the clinical T factor and clinical staging (c-Stage) in the latest UICC (8th version) of the TNM classification [8]. For solid nodules, the total size (TS) measurement, which is equal to the SS, has been adopted as before. On the other hand, a recent study by Hattori et al. indicated that the presence of a GGO component in the tumor was an index of favorable prognosis [9, 10].

In the present study, we retrospectively evaluated whether the clinical staging based on the 8th version of the UICC could reflect the postoperative prognosis of patients with small NSCLC. In addition, differences in prognoses between patients with solid- and subsolid-type lung cancers were verified.

## Methods

This retrospective study was approved by our Institutional Review Board, and written informed consent was waived (approval no. 2017–0437).

### Patient selection

Using the retrieval function of picture archiving and communication systems, we searched our institutional database for CT, positron emission tomography/CT, and pathological reports on patients with pathologically confirmed, surgically resected primary lung cancer between August 2010 and December 2013. During this period, 596 surgeries for lung cancer were performed in our institution. Based on the preoperative CT reports, radiologic information obtained from thin-section CTs included clinical TS, ratio of consolidation to tumor (C/T ratio), location of tumor, and c-Stage based on the TNM classification in the 7th version of UICC. Solid nodules were defined as those with a C/T ratio of 100%, whereas subsolid nodules were defined as those with a C/T ratio < 100%. Based on the TS and C/T ratios, the SSs of subsolid nodules were calculated, and the c-Stage was transferred from the 7th to 8th version definitions. Moreover, we selected NSCLCs measuring ≤3.0 cm in SS. Tumors that did not have information about the c-Stage were excluded from our cohort.

The clinical records of all selected cases were reviewed to obtain data on the patients’ age, sex, date of surgery, operative procedure performed, and postoperative course. Lobectomy was regarded as standard surgery, and wide wedge resection and segmentectomy were regarded as limited surgeries. The patients were scheduled for follow-ups every 1 to 3 months for 2 years after the surgery and every 6 months thereafter. In patients with a high risk of recurrence, a CT of the chest and abdomen was performed every 6 to 12 months, according to the physician’s recommendation. When recurrence was suspected, additional imaging surveys were performed.

### Statistical analysis

Disease-free survival (DFS) was assessed using the Kaplan–Meier method, and the survival curves for each group were compared using the log-rank test. DFS was defined as the interval between the surgery and the first disease recurrence, including local recurrence and distant metastasis, or death from any cause. In patients with multiple synchronous lung cancers, the tumor with the highest c-Stage was deemed to have relapsed. On univariate and multivariate analyses of DFS, the Cox proportional hazards model was used to assess the effects of tumor characteristics as potential prognostic factors.

Statistical analysis was performed using the commercial software SPSS version 23 (IBM Corp., Armonk, NY, USA). A *p*-value < 0.05 was considered statistically significant.

## Results

A total of 247 NSCLC cases in 231 patients (88 women and 143 men; average ± standard deviation of age, 67 ± 7 years) were included in this study. Sixteen patients had synchronous tumors. Thin-section CT findings included solid (*n* = 131) and subsolid (*n* = 116) nodules. Table 1 shows the correlation between the 7th and 8th version definitions of the c-Stage. The postoperative histopathologic diagnoses were adenocarcinoma (*n* = 203), squamous cell carcinoma (*n* = 28), adenosquamous carcinoma (*n* = 9), large-cell carcinoma (*n* = 5), mucoepidermoid carcinoma (*n* = 1), and adenoid cystic carcinoma (*n* = 1). Postoperative recurrence was observed in 30 patients. Tumor-related or unexplained deaths were observed in 18 patients. The other patient and tumor characteristics are shown in Table 2.

**Table 1:** The correlation of patients’ number between the 7th and 8th version of clinical TNM stage

		8th version				
		0	IA1	IA2	IA3	IB
7th version	IA	28	37	69	38	
	IB					71

**Table 2:** The other patient and tumor characteristics

	Number or Mean ± SD
Age (years)	67 ± 7
Female/ Male (*n*)	88/ 143
RUL/ RML/ RLL/ LUL/ LLL (*n*)	73/ 22/ 47/ 73/ 32
Wide wedge resection/ Segmentectomy/ Lobectomy (*n*)	29/ 50/ 168
Subsolid/ Solid nodule (*n*)	116/ 131
Adenocarcinoma/ SqCC/ ASC/ LCC/ Others (*n*)	203/ 28/ 9/ 5/ 2
Total Size (mm)	19.8 ± 6.2
Solid Size (mm)	14.9 ± 8.3
Interval between CT and surgery (days)	19 ± 16
Postoperative follow-up duration (months)	42 ± 19

*RUL* right upper lobe, *RML* right middle lobe, *RLL* right lower lobe, *LUL* left upper lobe, *LLL* left lower lobe, *SqCC* squamous cell carcinoma, *ASC* adenosquamous carcinoma, *LCC* large cell carcinoma

**Fig. 1:**
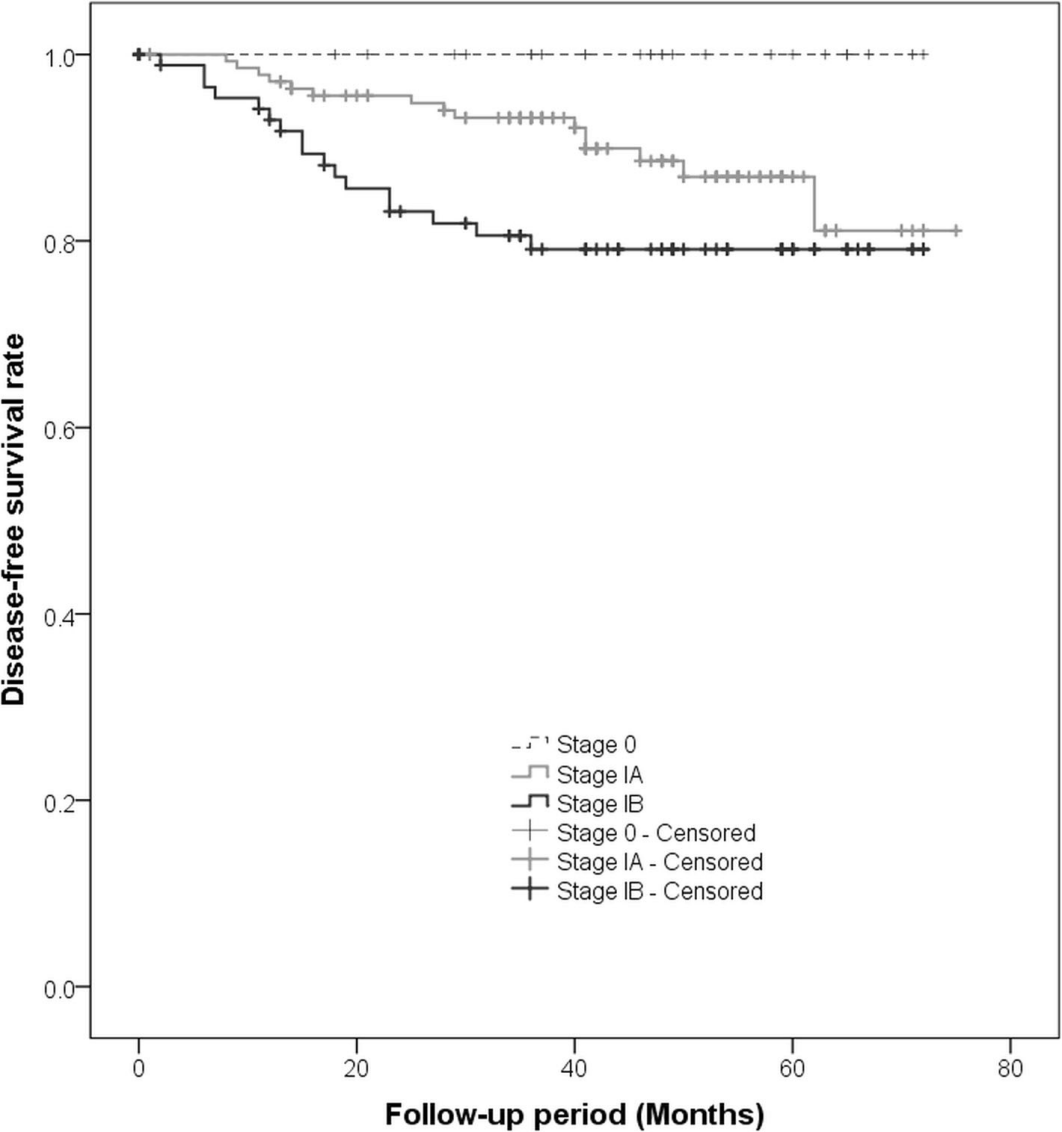
The graph shows Kaplan-Meier curves for disease-free survival according to clinical stage 0-IB. There is a significant difference among c-Stage 0, IA, and IB tumors (*p* = 0.016)

**Fig. 2:**
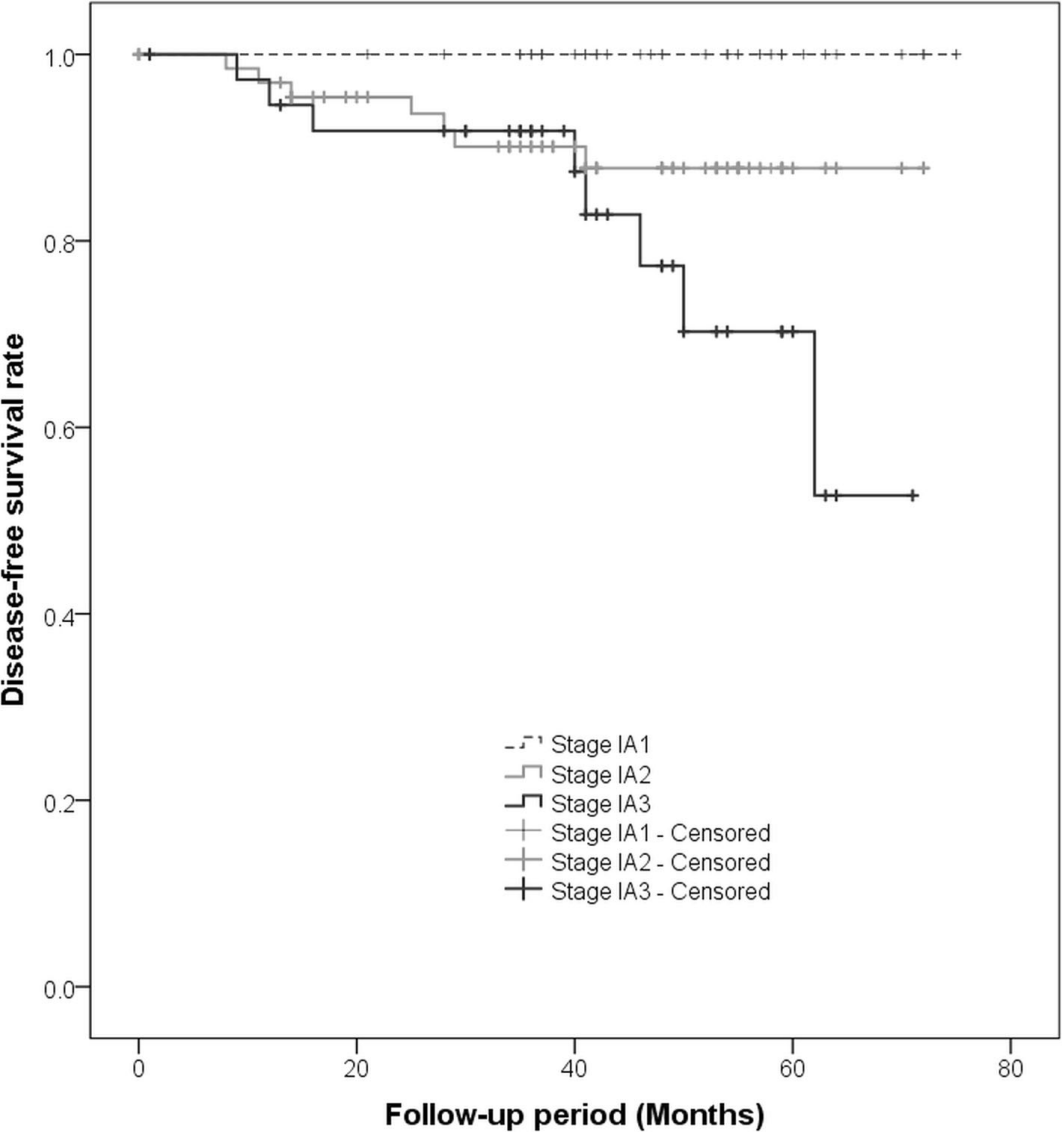
The graph shows Kaplan-Meier curves for disease-free survival according to the subgroup of clinical stage IA tumors. There is a significant difference among c-Stage IA1, IA2, and IA3 tumors (*p* = 0.011)

**Fig. 3:**
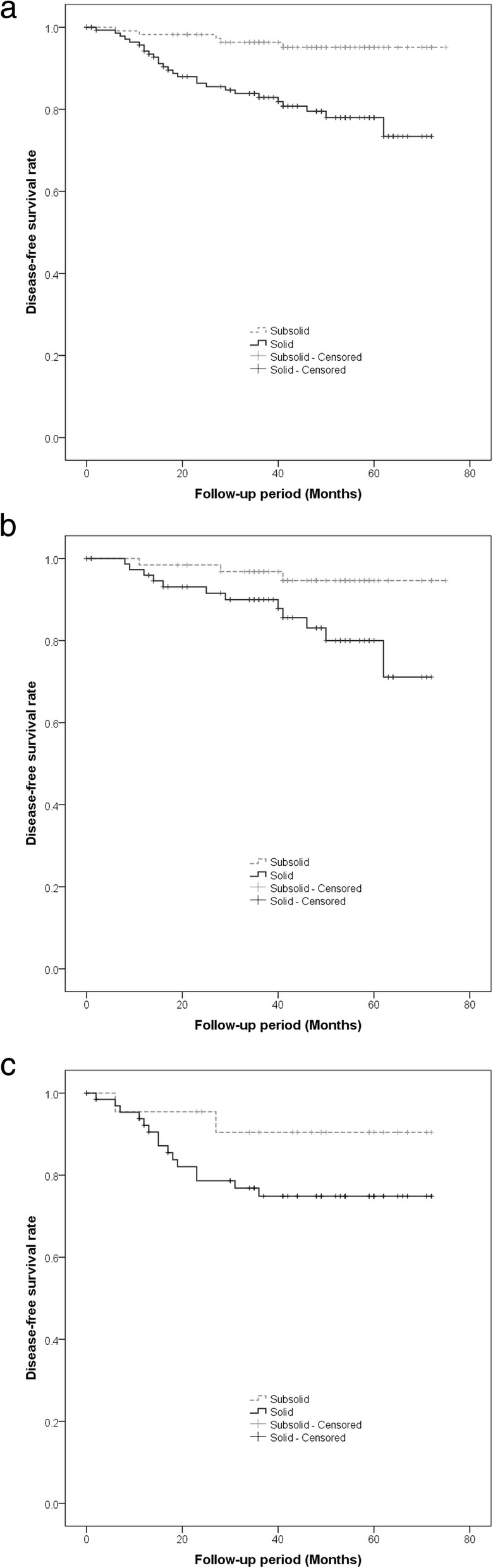
The graph shows Kaplan-Meier curves for disease-free survival according to the subgroup of solid tumors and subsolid tumors. For all tumors (**a**) and clinical stage IA tumors (**b**), patients with subsolid tumors show a significantly better prognosis than those with solid tumors (*p* < 0.001 and *p* = 0.026, respectively). For clinical stage IB (**c**), patients with subsolid tumors show a better prognosis than patients with solid tumors among the clinical stage IB tumors, although significant differences were not observed (*p* = 0.102)

Kaplan–Meier curves for DFS of all patients with NSCLC showed a significant difference among those with c-Stage 0, IA, and IB tumors (*p* = 0.016, Fig. 1), and patients with c-Stage 0 tumors showed a better prognosis than those with c-Stage IA and IB tumors. According to the subgroup of c-Stage IA tumors, there were significant differences among c-Stage IA1, IA2, and IA3 (*p* = 0.011, Fig. 2); patients with c-Stage IA1 tumors showed a better prognosis than those with c-Stage IA2 and IA3 tumors. In addition, patients with subsolid tumors showed a significantly better prognosis than those with solid tumors (*p* < 0.001, Fig. 3a). Patients with subsolid tumors showed a significantly better prognosis than patients with solid tumors among the c-Stage IA tumors (*p* = 0.026, Fig. 3b), and patients with subsolid tumors showed a better prognosis than patients with solid tumors among the c-Stage IB tumors, although significant differences were not observed (*p* = 0.102, Fig. 3c). Univariate and multivariate Cox proportional hazards models indicated that the patients’ age, c-Stage, and presence of solid nodules were significant factors that correlated with the postoperative prognosis, although the patients’ sex and operative procedures performed were not significant (Table 3). In the subgroup of adenocarcinomas, univariate and multivariate Cox proportional hazards models indicated that the c-Stage and presence of solid nodules were significant factors correlated with postoperative prognosis (Table 4).

**Table 3:** Univariate and multivariate Cox proportional hazards model for disease-free survival

	Univariate		Multivariate	
Factor	HR (95% CI)	*P* value	HR (95% CI)	*P* value
Age	1.059 (1.006–1.116)	0.029	1.055 (1.003–1.109)	0.038
Male (vs. female)	1.707 (0.781–3.730)	0.122	1.011 (0.451–2.266)	0.979
Limited surgery (vs. Lobectomy)	0.383 (0.133–1.098)	0.074	0.645 (0.223–1.868)	0.419
c-Stage	1.839 (1.305–2.591)	< 0.001	1.600 (1.078–2.376)	0.020
Solid nodule (vs. Subsolid nodule)	5.153 (1.972–13.467)	0.001	3.077 (1.111–8.521)	0.031

**Table 4:** Univariate and multivariate Cox proportional hazards model for disease-free survival in the adenocarcinomas

	Univariate		Multivariate	
Factor	HR (95% CI)	*P* value	HR (95% CI)	*P* value
Age	1.044 (0.980–1.113)	0.179	1.048 (0.984–1.117)	0.146
Male (vs. female)	1.729 (0.648–4.610)	0.274	1.101 (0.403–3.004	0.852
Limited surgery (vs. Lobectomy)	0.263 (0.060–1.148)	0.076	0.384 (0.088–1.674)	0.203
c-Stage	2.716 (1.561–4.727)	< 0.001	4.293 (1.628–11.318)	0.003
Solid nodule (vs. Subsolid nodule)	7.313 (2.116–25.274)	0.002	5.132 (1.452–18.143)	0.011

## Discussion

The present study showed that the 8th version of the UICC of the TNM classification based on SS measurements in subsolid lung cancers on a multidetector CT accurately correlated with postoperative recurrence in patients with c-Stage 0, IA, and IB NSCLCs. In patients with c-Stage IA, the TNM classification based on SS measurements accurately correlated with postoperative recurrence. The prognosis of patients with c-Stage IA1 tumors with SS ≤1.0 cm was as excellent as that of patients with c-Stage 0 tumors that were regarded as noninvasive tumors. The multivariate Cox proportional hazards model indicated that the c-Stage could independently predict the postoperative prognosis.

Our study also showed whether solid or subsolid nodules also correlated significantly with patient prognosis. In patients with c-Stage IA tumors with SS ≤3.0 cm, patients with subsolid nodules showed significantly better prognosis than that of patients with solid nodules. Our results are consistent with the results of a previous study by Hattori et al. that showed that the presence of a GGO component within a tumor indicates favorable prognosis in patients with c-Stage IA NSCLC [9, 10]. In c-Stage IB tumors with pleural invasion and/or SS > 3 cm, patients with subsolid nodules showed tendencies of a better prognosis than those of patients with solid nodules on the Kaplan–Meier curves, although significant differences were not observed. Multivariate analysis demonstrated that the presence of solid/ subsolid nodules was a significant independent factor for DFS, as with age and c-Stage. Subsolid nodules are mainly composed of adenocarcinomas, in which GGO reflects the replacement of the alveolar epithelium by well-differentiated tumor cells. On the other hand, solid nodules include squamous cell carcinomas and large cell carcinomas, besides adenocarcinomas. Since squamous cell carcinomas, adenosquamous carcinomas, and large cell carcinomas are more invasive and malignant than adenocarcinomas, these histopathological types might lead to a worse prognosis in patients with solid nodules [1, 11–14]. Therefore, we surveyed only adenocarcinomas and found that the c-Stage and presence of solid/subsolid nodules were significant factors for DFS, even within the subgroup. The presence or absence of a GGO component could have a strong influence on the prognosis, even in patients with adenocarcinoma.

A recent study proved that the concordance rate between the c-Stage and pathological stage (p-Stage) of the 7th version of the UICC for small lung cancers had moderate reproducibility, and p-Stage but not c-Stage was a significant prognostic indicator of DFS upon multivariate analysis [15]. For c-Stage I NSCLCs, stereotactic radiotherapy (SRT) has been recently shown to confer outcomes comparable with those of surgery [16, 17]. However, in this setting, p-Stage is not confirmed. From our results, to predict the prognosis of patients with unoperated NSCLC, both the c-Stage and the presence of either solid/ subsolid nodules should be considered.

This study had several limitations. It was a retrospective and a single-center study, the TSs were measured manually, and the SSs were calculated on the basis of the TSs and C/T ratios described in the preoperative radiological reports. Hence, solid nodules were differentiated visually from subsolid nodules by radiologists. This might have led to some measurement errors. If computer-aided diagnosis software could automatically discriminate solid from subsolid nodules and measure the maximal diameter of the solid components in tumors, then the measurement accuracy could be improved. In addition, the volume of the solid component of subsolid NSCLCs shows better correlation with postoperative recurrence [4, 18]. Moreover, the pathological invasive size was not measured and compared with SS in this study because our cohort included old cases, and the pathologists at the time did not measure this.

## Conclusions

In conclusion, for early-stage NSCLCs, the c-Stage of the 8th version of the UICC based on the SS in a subsolid tumor was useful for predicting the postoperative DFS. On the other hand, whether solid or subsolid nodules were present was another independent prognostic factor. Therefore, to evaluate the prognosis of patients with unoperated NSCLC more accurately, both the c-Stage and the presence of either solid/ subsolid nodules should be considered.

## Data Availability

The dataset supporting the conclusions of this article is included within the article. The datasets used and/or analyzed during the current study are available from the corresponding author upon reasonable request.

## Abbreviations

C/T ratio: Ratio of consolidation to tumor
c-Stage: Clinical stage
CT: Computed tomography
DFS: Disease-free survival
GGO: Ground-glass opacity
NSCLC: Non-small cell lung cancer
p-Stage: Pathological stage
SS: Solid Size
TS: Total Size
UICC: Union for International Cancer Control
